# EARLY TIME RESTRICTED FEEDING IMPROVES HIGH DENSITY LIPOPROTEIN FUNCTION IN GERIATRIC MONKEYS

**DOI:** 10.1093/geroni/igz038.389

**Published:** 2019-11-08

**Authors:** Kylie Kavanagh, Alexander Bashore, Matthew Davis, Chrissy Sherrill, John Parks

**Affiliations:** Wake Forest School of Medicine, Winston Salem, North Carolina, United States

## Abstract

Ageing conveys the greatest risk for cardiovascular disease (CVD) development, which is the dominant cause of mortality in developed nations. High density lipoprotein (HDL) particles mediate reverse cholesterol transport, are anti-inflammatory and their function predicts CVD. We observed lower plasma cholesterol efflux capacity in geriatric vervet monkeys (p=0.03) when consuming either healthy or Western diets. Adult (n=16) and geriatric (n=19) monkeys were stratified into groups fed Western diet on ad libitum (Ad Lib) or early time restricted feeding (eTFR) schedules. eTRF supplied excess food only between 6am to 2pm. Housing, seasonality and fasting conditions for data and sample collections were equivalent. After 6 months, cholesterol efflux to HDL was greater in eTRF monkeys (p=0.01), with no age by group interaction. Efflux media and plasma was chromatographically separated to confirm labelled cholesterol, and enzymatically measured cholesterol, respectively, was affiliated with HDL particles. eTRF monkeys had higher total plasma cholesterol levels (p=0.03) which was due to greater cholesterol amounts associated with only HDL, and resulted in HDL particles that were larger. eTRF resulted in robustly better HDL function such that measures from geriatric individuals were comparable to younger adults. Additionally, no differences in adiposity was observed in eTRF monkeys. Few interventions are known to raise HDL levels, and more importantly, are confirmed to improve HDL function. Our study is to date the largest, longest, controlled eTRF evaluation in primates and we show that positive biological effects are observable in HDL isolated from both adult and geriatric individuals independently of weight change.

